# Modelling allocation of resources in prevention and control of obstetric fistula in Ugandan women

**DOI:** 10.1371/journal.pone.0238059

**Published:** 2020-09-10

**Authors:** Betty Nannyonga, Martin Singull

**Affiliations:** 1 Department of Mathematics, School of Physical Sciences, College of Natural Sciences, Makerere University, Kampala, Uganda; 2 Department of Mathematics, Mathematical Statistics, Linköping University, Linköping, Sweden; University of Mississippi Medical Center, UNITED STATES

## Abstract

In spite of reliable and skilled healthcare resources, the prevalence rate of obstetric fistula in Uganda is high. The risk factors for obstetric fistula cut across due to high poverty rates and cultural barriers. The main objective of this study was to assess the impact of inability to access skilled healthcare at delivery and implications to the economy. The specific objective was to determine the best way of investment in getting women access to skilled healthcare before, during and after child birth. The question to be answered was whether it was more economical to invest in getting women access to skilled healthcare, or in expanding healthcare. The study was conducted using data from the Uganda Demographic Health Survey 2016. The data was from 18,506 women in the age group of 15-49 in 15 regions around the country. Results show that the highest investment in providing access to skilled healthcare is required when there are few skilled healthcare centres. On the other hand, if there is little investment in providing access to skilled healthcare during child birth, many skilled healthcare centres are required. Results show further that the minimum time taken to reduce fistula prevalence is attained when there are many women accessing skilled healthcare in the few equipped health centres. However, if there are many skilled healthcare centres but a few women treated for obstetric fistula, then it will take longer to reduce fistula prevalence. Fitting the model to data suggested that Uganda has a big backlog of women to treat for obstetric fistula as in all skilled healthcare centres, there were less women treated than expected. Although still under the expected figure, the benefit of these treatments for obstetric fistula is that for every one woman treated, 8 more would seek treatment for the condition. This would however cost the country a great deal in that the treatment funds would perhaps give more returns if diverted to outreach activities aimed to get women seek skilled healthcare during child birth.

## Introduction

Obstetric fistula is a medical condition in which a fistula (hole) develops between either the rectum and vagina (rectovaginal fistula), or between the bladder and vagina (vesicovaginal fistula) [1, 2]. It is usually caused by prolonged, obstructed labour, without timely medical intervention—typically an emergency caesarean section. During unassisted, prolonged and obstructed labour, the sustained pressure of the baby’s head on the mother’s pelvic bone damages soft tissues, creating a hole-or fistula-between the vagina and the bladder and/or rectum [2]. An obstetric fistula leaves a woman incontinent of urine or faeces or both.

Prolonged obstructed labour is the direct cause of obstetric fistula [1]. However, there are indirect causes some of which are social, political, and/or economical [3]. Indirect causes concern issues of poverty, malnutrition, lack of education, early marriages and childbirth. The role and status of women in developing countries, harmful traditional practices, sexual violence, and inability to access skilled healthcare [4–6] are also indirect drivers of obstetric fistula. Although Uganda has reliable and skilled healthcare resources, most women do not access skilled maternal care during pregnancy. In some instances, it is due to lack of transport, and/or long distances to the healthcare facilities. The women also lack support from their spouses, and at times have to get permission. In cultural settings, permission implies the husband providing the required fare to the healthcare facility, or avoiding a domestic dispute if you dared go without approval from the husband. Due to these reasons, the women opt for local and/or traditional birth attendants. Although in some cases delivery is successful, majority of fistula cases occurred in such settings. During obstructed labour, women need emergency caesarean section which is not available in local or traditional birth settings. In such cases, 69% of the neonatal outcomes are still birth, 2% are early neonatal death [7], and only 9% live births. In addition to losing their child, the affected women are both physically and socially disabled [8–14], humiliated [2, 3, 15–17], endure recurrent vaginal or urinary tract infections [3, 17, 18], and chronic incontinence [17, 19–21]. Treatment of fistulas is by surgery, which is often unaffordable to the impoverished women [22].

The current lifetime prevalence of vaginal fistula symptoms in Uganda is between 16.3–22.5 per 1000 women of reproductive age, i.e., 15–49 [23]. This is equivalent to 1.63–2.25 percent [24, 25], putting Uganda highest among African countries [23]. To reduce the prevalence of fistula in Uganda, 29 fistula centres were set up, distributed in 24 districts around the country [22]. Among them, Kitovu Hospital, hosts ‘Fistula Camps’ four times a year treating between 60 –100 women [26–28]. Obstructed labour is an important cause of maternal deaths in poor communities in which there is no easy access to functioning health facilities [29]. Tackling the problem of obstructed labour requires the ability to access adequately equipped and skilled healthcare facilities whenever problems arise in labour. In this study, we hypothesise that availability of skilled healthcare services could reduce the prevalence of obstetric fistula in Uganda. The causal link between lack of skilled healthcare to obstetric fistula development is studied. We seek to answer whether it is plausible to invest in increasing the number of women who access skilled healthcare or those who get treatment after development of obstetric fistula. The dynamic model designed has three variables:- women with fistula, those treated, and the recovered.

## The dynamic model

The model used in this study has three classes: Women with fistulas *F*, those treated *T*, and the recovered, *R*. We hypothesise that if women do not get access to skilled healthcare during obstructed labour, then they develop obstetric fistula. Let the proportion of funds available fro treatment be *p*_*a*_, and for providing women access to skilled healthcare during child birth be 1 − *p*_*a*_. Let *π* be the rate at which the funds for skilled healthcare are availed. Then, the rate of development of obstetric fistula *β* is dependent on access to available funds, i.e., *β* ∝ (*p*_*a*_, *π*). If the rate of getting women access to skilled healthcare is high, then we predict low fistula development; i.e., ${\text{lim}}_{p_{a}\to 1}\beta \approx 0$, while ${\text{lim}}_{p_{a}\to 0}\beta \approx 1$. When women develop fistula, some of them die from related causes while others get access to treatment. Let the respective fistula related death and treatment rates be *δ*_*f*_ and *γ*. Treatment is dependent on the cost per unit operation and the total available funds. Let the point where at least 50% of fistula symptomatic women get access to treatment be the half saturation constant *η*. Therefore, with the previous hypothesis, the rate at which women get treatment tends to zero whenever *p*_*a*_ → 0; that is, ${\text{lim}}_{p_{a}\to 0}\gamma \approx 0$. That is, if all funds are invested in getting women access to skilled healthcare, then no funds will be available for treatment. This study therefore determines the best way to invest in providing women access to skilled healthcare before, during and after child birth. We find the best economic strategy of investment to reduce women who do not access skilled healthcare, and those who develop fistula due to obstructed labour. We predict whether the government should invest more in access to or expand skilled healthcare.

To achieve our objectives, we set up constraints aimed to reduce fistula prevalence as a function of funds invested. The constraints applied are intended to

1. invest a proportion *p*_*a*_ ∈ [0, 1] directly in treatment, and
2. indirectly invest 1 − *p*_*a*_ in providing women access to skilled healthcare.

These constraints imply that increase in access to fistula treatment is $\frac{\gamma p_{a}}{\eta +p_{a}}$, and the corresponding baseline reduction in fistula development is $\frac{\beta (1-p_{a})}{\pi +(1-p_{a})}.$ These functions imply diminishing returns in investment either directly in providing access to skilled healthcare, or in providing treatment. The parameter *η* determines the level *p*_*a*_ must be set to in order to achieve 50% of maximal decrease in prevalence of obstetric fistula. Thus larger values of *η* mean that it is more difficult to achieve a reduction in fistula cases through direct investment in providing access to skilled healthcare. Similarly, larger values of *π* mean that it is more difficult to achieve decreases in the number of cases of obstetric fistula through direct investment in treatment.

Upon successful treatment, some women recover [27, 28] while others do not [30]. Let the recovery rate be *ϕ*, and the proportion of women who do not recover be *q*. Therefore, women who do not recover go back to class *F* while those who recover (the 1 − *q*), join class *R*. The successfully operated women remain fistula-free for the remainder of their lives, for as long as they follow medical advice [30]. Women who do not follow medical advice are susceptible to reoccurrence of fistula (i.e., move back from *R* to *F*). Let the rate at which women redevelop fistula be *σ*. Redevelopment of fistula depends on the number of times women fail to adhere to medical advice. Let the number of times women fail to adhere to medical advice be *κ*. To weight the transition rate from *R* to *F*, we make it a function *κ*. We seek a function of *κ*, i.e., *f*(*κ*), that is strictly increasing and satisfies *f*′(0) = 1, *f*(0) = 0, *f*(∞) = 1. Such a function can be $f\left(\kappa \right)=\text{tanh}\left(\kappa \right)=(1-\frac{2}{e^{2\kappa }+1})\in \left[0,1\right].$ Note that when all women adhere to medical advice after surgery, *κ* = 0. Using our function, we see that lim_*κ*→0_ *f*(*κ*) = 0. This implies that all treated women do not redevelop fistula if they adhere to medical advice.

**Fig 1:**
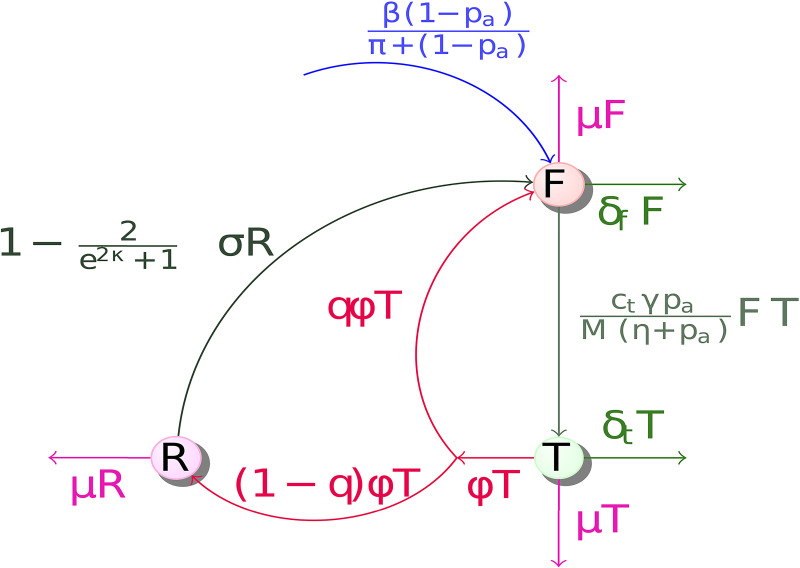
Dynamics of women with obstetric fistula in presence of skilled healthcare.

Let *c*_*t*_ be the unit cost of surgery per woman and *M* the total available funds. Then, $\frac{c_{t}}{M}F$ is the actual number of women treated for their fistulas. Let also *δ*_*T*_ be the death rate during treatment, and *μ* the per capita natural mortality rate. Then, from the definitions, descriptions and assumptions, we have the flux digram in Fig 1, and the corresponding model in Eq (1).

$$\begin{array}{cc} \frac{dF}{dt} & =\frac{\beta (1-p_{a})}{\pi +(1-p_{a})}-\frac{c_{t}\gamma p_{a}}{M(\eta +p_{a})}FT+q\phi T+(1-\frac{2}{e^{2\kappa }+1})\sigma R-\left(\delta_{f}+\mu \right)F,\\ \frac{dT}{dt} & =\frac{c_{t}\gamma p_{a}}{M(\eta +p_{a})}FT-\left(\phi +\delta_{t}+\mu \right)T,\\ \frac{dR}{dt} & =\left(1-q\right)\phi T-[(1-\frac{2}{e^{2\kappa }+1})\sigma +\mu ]R. \end{array}$$ (1)

All parameters of the model are positive. Since the state variables of the autonomous model (1) are non-negative for all time *t*, the model can be studied in the invariant region:

$$\begin{array}{c} G=\{\left(F,T,R\right)\in I\;R^{3}:F,T,R\geq 0\}, \end{array}$$

where the model is mathematically well-posed. We analyse the system in Eq (1) for existence, stability and how to optimise investment into reducing prevalence of obstetric fistula.

## Available data

The data used in this study was collected by the Uganda Bureau of Statistics in the Demographic Health Survey. All the sample data collected was used and the demographic data of the participants represent the greater population. Ethical approval for secondary data analysis was not required since the data was already coded and did not bear names nor addresses of the participants. Data was grouped into regions and age and observed patient protection and confidentiality. The participants were women in the reproductive age group 15–49 in 15 different parts of the country namely South Central, North Central, Kampala, Busoga, Bukedi, Bugisu, Teso, Karamoja, Lango, Acholi, West Nile, Bunyoro, Tooro, Kigezi and Ankole. These areas were randomly selected from the four Ugandan regions. The total number of hospitals (public and private) in Uganda is 155 [22]. Of these 2 are National Referral Hospitals (Mulago and Butabika), 14 are Regional Referral Hospitals (RRHs) and 139 are General Hospitals (GHs). The current population of Uganda is 43,538,146 as of Tuesday, November 27 2018, based on the latest United Nations estimates [31]. The fertility rate is 5.82, and mean age at first birth 18.2 [23]. Only 58.3% percent of Ugandan women receive the recommended four antenatal care visits. The life expectancy at birth for Ugandan women is 56.9 years [32, 33] and between 140,000 and 200,000 Ugandan women in the reproductive age group (14–49 years) have a fistula [34]. Overall, one out of 50 women has had fistula [35]. Currently, fistula operations are carried out free of charge in 29 hospitals, from 24 districts [1] (i.e., Kitovu hospital in Masaka, Arua Regional Referral Hospital in Arua, Church Of Uganda—Kisiizi Hospital in Rukungiri, Fort Portal Regional Referral Hospital in Fort Portal, Hoima Regional Referral Hospital in Hoima, Kabale Regional Referral Hospital in Kabale, Kagadi Hospita in Kibaale, Kagando Hospital in Kagando Kasese, Kamuli Mission Hospital in Kamuli, Kiboga Hospital in Kiboga, Kisoro Hospital in Kisoro, Kitwe Health Center IV in Ntungamo, Kumi Hospital in Kumi, Mbale Regional Referral Hospital in Mbale, Mityana Hospital in Mityana, Mulago National Referral and Teaching Hospital in Kampala, Soroti Regional Referral Hospital in Soroti and St. Mary’s Hospital Lacor in Gulu, Virika Mission Hospital in Fort Portal, CoRSU Hospital in Kisubi Entebbe). Fortunately, about 80% of all the operations are successful [26]. A fistula repair operation costs about $400 [26]. In 2015, the fistula treatment clinic at Kitovu hospital treated nearly 100 women over eleven days [1] and for every woman who received treatment for her fistula, at least 50 more went without [1].

### Parameter estimation

In this section we estimate parameters to fit the model using available data. The rate at which women develop fistula *β* is estimated using the prevalence rate of fistula in the interviewed women. Of the total of 18,506 women in the study, approximately 1.6319% had ever had fistula symptoms. This is equivalent to 16.319 per 1000 women, and compares well with the current average lifetime prevalence of vaginal fistula symptoms in Uganda, which is 19.2 per 1000 women of reproductive age (15-49) [23]. Prevalence can be transformed into a rate when we divide it by the average length of time women spend in obstructed labour (2.25 days [7]). This implies that *β* = 16.319/1000 × 365/2.25 = 2.647 percent per year. The proportion of funds invested in treatment ranges from 0 (zero funds available) and 1 (funds for operation of one woman are available), i.e., *p*_*a*_ ∈ [0, 1]. From the data, 302 women had ever had fistula symptoms, therefore the value of *π* where at least 50% of them would not have developed fistula is 151. If not treated, women may die from fistula related complications at a rate equivalent to the reciprocal of the duration these women spend with fistula without surgery. The average age at which women have fistula is 30.8 years [23], and the life expectancy at birth is 56.9 years [32, 33]. Then, the expected time with which the women live with fistula is 56.9-30.8 = 26.1 years giving fistula death rate per year *δ*_*f*_ = 1/26.1 = 0.0383 = 3.83 percent per year.

**Fig 2:**
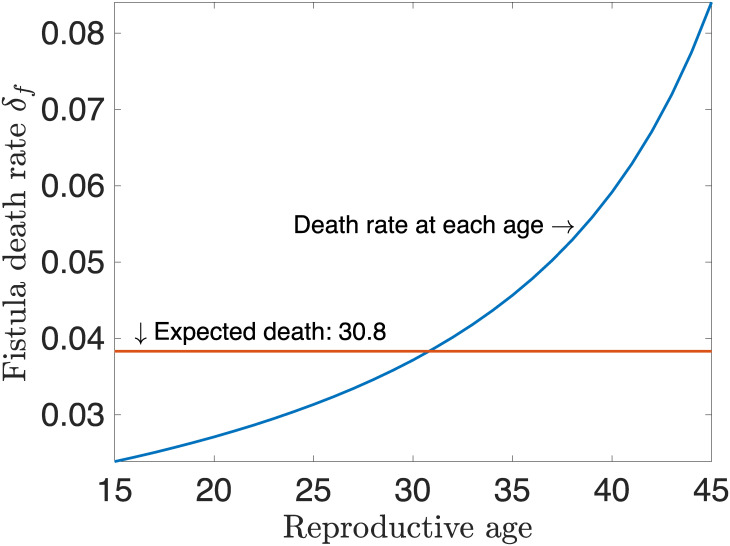
Plot of age of the susceptible women to the corresponding fistula related death rates *δ*_*f*_.

Fig 2 shows the variation of death with age in women of reproductive age. The figure shows that women were more likely to die from fistula related complications as they got older. The rate at which women are treated is obtained by getting the proportion of women treated per year divided by the length of time these women live with fistula in years. This implies that *γ* = 18238/(417750 × (56.9 − 30.8)) = 0.0017 per year. Let *c*_*t*_ (= USD 400 [26]) be the unit cost for treatment for one fistula woman and *M* be the total funds available for one fistula camp. Define *η* as the half saturation constant, which is the point at which women from at least 50% of Uganda’s hospitals that provide fistula operations, received surgery. According to the facility and treatment information, 29 hospitals provide fistula operations [1, 36], giving *η* = 50% × 29 = 14.5. Fistula related death rates during operations can be obtained using the percentage of women who die in the process (i.e., 20%) [26]. Assuming that they die on the day of operation due to complications during surgery, then *δ*_*t*_ = 0.2 per year. The recovery rate *ϕ* is dependent on how severe the fistula was, but usually after 1-week, the operated woman can return to work [37]. Therefore, we can set *ϕ* = 0.0192 per year.

The proportion of women *q* who do not recover after an operation can be estimated to be 0.08 [38]. The other proportion 1 − *q* joins the recovered class where they remain if they follow medical advice. When they do not, women can develop fistula again at a rate *σ* which is weighted depending on how many times women fail to follow medical advice *κ*. For example, the mean age for women with fistula in Uganda is 30.8 [23], and the average life expectancy for Ugandan women is 56.9 [32]. Assuming that women are operated at age 30.8, they will live approximately 26 years of life after fistula. If we assume that they would have followed up twice per year, then maximum *κ* would be 52. We could equate *σ* to *β* or use a modification parameter that either enhances or inhibits *σ* in relation to *β* depending on how likely a recovered woman can get fistula again [38]. The per capita natural mortality rate of female Ugandans is given by *μ* = 1/56.9 = 0.0176 where 56.9 is the life expectancy [32]. These parameter estimates are summarised in Table 1. We can now analyse the model, fit the estimated parameters, perform regression, and determine the threshold values that can reduce fistula prevalence in Uganda.

**Table 1:** Parameter estimates and confidence intervals.

Parameter description	Symbol/ Value		Units
Percent investment in antenatal care	*p*_*a*_	[0—1]	
Cost per fistula operation	*c*_*t*_	400	USD
Half saturation constant (antenatal)	*π*	151	Women
Fistula development rate	*β*	2.647	%Per year
Number of times women fail to adhere to medical advice	*κ*	0—52	Per year
Fistula reoccurrence rate	*σ*	*f*(*β*)	% Per year
Per capita female mortality rate	*μ*	1.76	% Per year
Fistula treatment rate	*γ*	0.17	% Per year
Proportion of operation failure	*q*	8	% Per year
Death rate due to fistula	*δ*_*f*_	3.83	% Per year
Death rate during operation	*δ*_*t*_	20	%Per year
Recovery rate	*ϕ*	1.92	% Per year
Half saturation constant (treatment)	*η*	14.5	Treatment centers

## Analysis of the model

The model will be analysed for existence of steady states and stability. Two steady states are obtained: the initial state when there are fistula women but no surgery, and the state with surgery interventions.

### The treatment-free equilibrium point $A_{0}$

This is the state when there is no treatment for symptomatic women. At this point both *T* and *R* are zero giving

$$\begin{array}{c} A_{0}=\{F_{0}^{*}=\frac{\beta (1-p_{a})}{\left(\delta_{f}+\mu \right)\left(\pi +\left(1-p_{a}\right)\right)},\;\;T=0,\;\;R=0\}, \end{array}$$ (2)

which exists for all parameter values since 0 ≤ *p*_*a*_ ≤ 1. It is observed that ${\text{lim}}_{p_{a}\to 1}F_{0}=0$. Therefore, with 100% investment in skilled healthcare during child birth, there are no initial number of women with fistula. This would imply that if we get pregnant women access to skilled healthcare during delivery, then no woman would develop obstetric fistula. Also note that $\frac{\partial F_{0}}{\partial p_{a}}<0,$ agreeing with the prior result that increasing investment into access to skilled healthcare reduces the number of women with fistula symptoms. Recall that *π* determines the level *p*_*a*_ must be set to have a 50% reduction in fistula development due to lack of skilled healthcare during child birth. It is therefore paramount to study the behaviour of *p*_*a*_ with varying values of *π* and *η* to determine plausible investment strategies and also how long it would take to reduce fistula women to less than 20.

**Fig 3:**
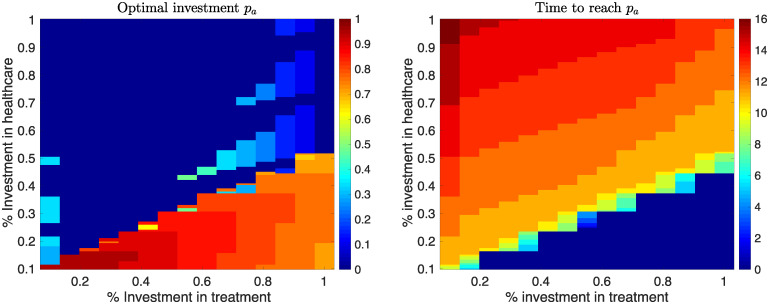
Optimal values of investment strategies *p*_*a*_ and the time it takes to attain a reduction in fistula cases to less than 20 per year while varying the percent efforts to get women access to skilled healthcare during child birth. Other parameter values used are in Table 1.

Fig 3 gives the different scenarios of investment to prevent development of obstetric fistula in pregnant women when skilled healthcare is available. The figure is obtained by varying the percent effort required to get women access to skilled healthcare *η*, and effort to get them treatment *π*.. Corresponding values of optimal values of *p*_*a*_ required to reduce fistula women to less than 20, $p_{a_{optimal}},$ and the minimum time required to attain this value are shown in the figure and a random sample in Table 2.

**Table 2:** Values of % effort in providing access to skilled healthcare *η*, and the % effort in treatment *π* to reduce fistula women to less than 20 with the corresponding $p_{a_{optimal}},$ and the time to reach the optimal investment strategy.

*η*	29	29	25	23	20	15	12	11	7	3
*π*	151	16	84	113	151	132	103	54	45	16
*p*_*a*_	0	0	0	0.2857	0.4286	0.619	0.7143	0	0.8571	0.9524
Time to reach *p*_*a*_ (years)	10	14	11	10	9	8	8	10	9	8

From Fig 3 and Table 2, it is observed that the minimum time taken to reduce fistula women to less than 20 is attained when the number of hospitals that offer fistula treatment (*η*) is small but the number of women treated in these hospitals (*π*) is high. The longest time taken to reduce fistula women to less than 20 is obtained when the number of hospitals that offer fistula treatment is large but the number of women treated in these hospitals is low. Similarly, when little investment in access to skilled healthcare is done, to reduce fistula women to less than 20 requires large many women treated in a few hospitals. However, when both the number of hospitals that offer fistula treatment, and the number of women treated are small, then the highest investment in getting women into healthcare should be invested.

We also look at the effects of various investment strategies on the threshold parameters that describe fistula prevalence in the population. These thresholds give the number of women who develop obstetric fistula without access to skilled healthcare $R_{A},$ treatment $R_{T},$ and both $R_{0}.$ $R_{0}$ is given by

$$\begin{array}{c} R_{0}=\frac{c_{t}\gamma \left(1-p_{a}\right)}{M\left(\phi +\delta_{t}+\mu \right)\left(\eta +\left(1-p_{a}\right)\right)}\frac{\beta (1-p_{a})}{\left(\delta_{f}+\mu \right)\left(\pi +\left(1-p_{a}\right)\right)}=R_{T}\times R_{A}. \end{array}$$ (3)

**Fig 4:**
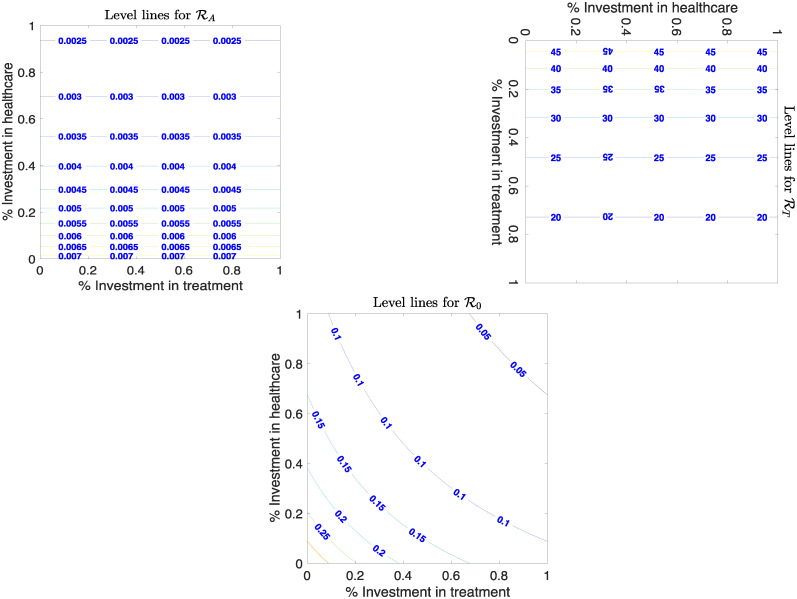
Change in threshold number of women who develop obstetric fistula with 50% investment (i.e., *p*_*a*_ = 0.5. The figure shows the corresponding changes in the number with access to skilled healthcare $R_{A},$ treatment $R_{T},$ and to both $R_{0}$. Other parameters values are given in Table 1.

The effects of varying efforts to get women into antenatal care *π* and treatment *η* to these threshold values are studied. For detailed expressions and descriptions of these functions refer to the Appendix. Fig 4 shows how the number of women treated and the number of hospitals affect these threshold values. We see in Fig 4 that with 100% access to skilled healthcare, no women develop fistula. However, lack of skilled healthcare causes high cases of fistula. When provision of skilled healthcare is at 100%, there are fewer cases of fistula development. These results show that there must be a balance between how many skilled healthcare centres available, and the women who access them. This can greatly reduce prevalence of obstetric fistula in Uganda.

### Stability in presence of treatment

**Fig 5:**
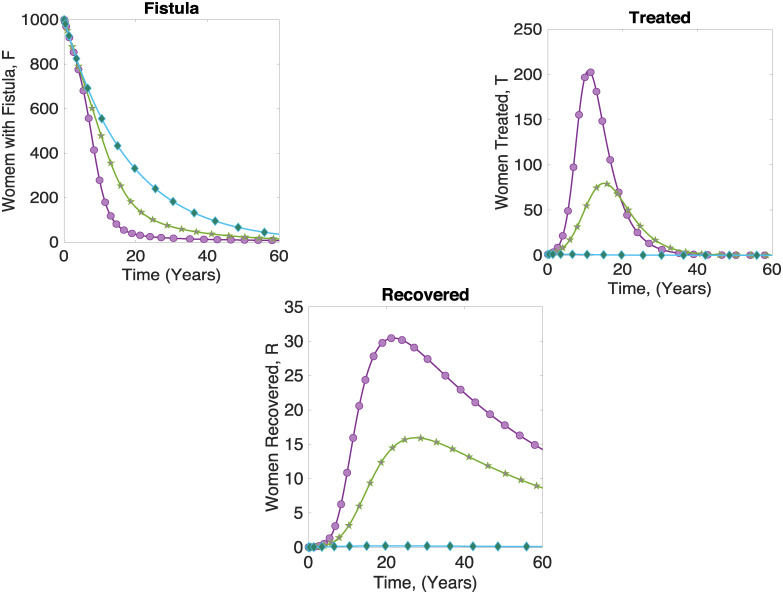
Evolution of fistula in a population with access to skilled healthcare and treatment; circled purple line *p*_*a*_ = 0.2, stars green line *p*_*a*_ = 0.5, and diamond blue line *p*_*a*_ = 0.9.

To study the behaviour of the system in presence of treatment, the model is simulated with initial values of *F*_0_ = 500, *T*_0_ = 1 and *R*_0_ = 0. The values of *p*_*a*_ used are 0.2, 0.5 and 0.9. It is observed from Fig 5 that when 20% of the funds are invested in providing access to skilled healthcare (i.e., when *p*_*a*_ = 0.2,) there is 80% investment in treating symptomatic women. Therefore, both *T* and *R* increase. As more is invested in access to skilled healthcare (i.e., 50%), there is slightly more women with fistula and less treated and recovered. When 90% is invested in providing access to skilled healthcare, (blue diamond line) there are many women with fistula and very few treated. This result implies that there must be a trade-off between providing access to skilled healthcare and treatment.

## Regression analysis

To determine the number of treated cases in relation to the total affected women, we use the eigenvalues of Jacobian (2) in the Appendix. We can thus write

$$\begin{array}{c} (\begin{array}{c} S^{\prime }\\ F^{\prime }\\ T^{\prime }\\ R^{\prime } \end{array})=A(\begin{array}{c} S^{\prime }\\ F^{\prime }\\ T^{\prime }\\ R^{\prime } \end{array})\;\;\text{and}\;\text{try}\;\text{a}\;\text{solution}\;\text{of}\;\text{the}\;\text{form}\;(\begin{array}{c} S\\ F\\ T\\ R \end{array})=re^{\Lambda t}, \end{array}$$ (4)

where *r* is the eigenvector associated with eigenvalue Λ. Thus,

$$\begin{array}{c} F\left(t\right)=F_{0}e^{(\delta_{f}+\mu )t},\;\;T\left(t\right)=T_{0}e^{\left(\phi +\delta_{t}+\mu \right)\left(R_{0}-1\right)t},\;\;\text{and}\;\;R\left(t\right)=e^{[(1-\frac{2}{e^{2\kappa }+1})\sigma +\mu ]t}, \end{array}$$ (5)

with $R_{0}$ defined as in Eq (3). We can now perform regression on Eq (5) by fitting it to data and estimate $R_{0}$, the threshold number of women expected to be treated for each one treated woman that joins the population of fistula women, and *T*_0_, and the initial number of women treated.

**Fig 6:**
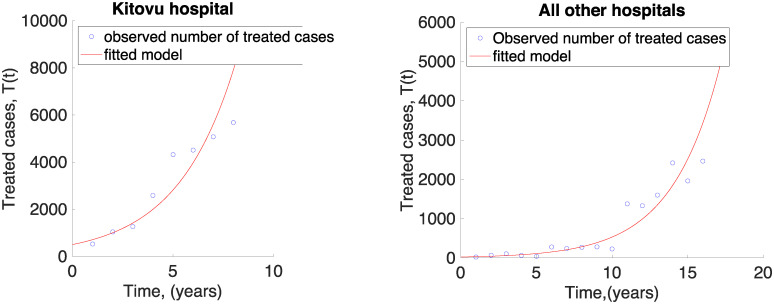
Linear regressions on the model using Kitovu data and all other hospitals data.

The data used for Kitovu hospital had year 1 as 2009, therefore the fit is for 2009-2016. Fitting is done using *R* and Matlab softwares. The result is shown in Fig 6. From the fit the model estimates that for each one women treated, 8 more are expected to be treated from Kitovu hospital. The model also estimates an initial number of treated women to be 506, and final 9,396. Therefore in 2016, 9,396 would have been treated. From the data, 5,680 were recorded to have been treated from Kitovu hospital. The data from all other hospitals was for the years from 2000 to 2015. This data estimates that for each women treated from all other fistula centres, 7 new ones will be treated. The initial number of women to have been treated from the centres is 25, while it predicts the number that should have been treated in 2015 to be 3816, a number that is 1,351 higher than the actual treated. Each of these treatments cost $400 [26]. Hence, the results show that for each woman treated, on average, 8 more would be treated. That is, for each $1 spent treating women, Uganda will need $8 more for treatment of fistula cases each year. In addition, for every one women treated, 50 go without treatment [1]. This implies that the ratio of no-treatment to treatment is 50/8 ≈ 7 women. Thus, Ugandan hospitals are getting $1 when they actually need $7. That it, they are getting 7 times less funds that they should to treat all the women. Our next question would then be how long would it take Ugandan hospitals to clear all the backlog of women who have not been able to get treatment. What would be the ideal thresholds each year.

## Discussion and conclusions

Healthcare services during pregnancy and childbirth are important for the survival and well-being of both the mother and the infant. Maternal health is a core dimension of the global health development agenda. The Government of Uganda prioritised maternal health in the national health agenda through the Roadmap for Accelerating the Reduction of Maternal and Neonatal Mortality and Morbidity in Uganda (2007-2015) and as a strategic and priority healthcare intervention area under the current Health Sector Development Plan (HSDP 2015/16-2019/20). Skilled birth attendants in Uganda play a vital role in providing comprehensive care for mothers and newborn infants, including preventing and managing obstetric complications. They are instrumental in supporting delivery, early postnatal care, prompt detection of problems, appropriate referrals, and actual management of mothers and newborn infants with danger signs. Skilled assistance during delivery is birth delivered with the assistance of doctors, nurses/midwives, and/or medical assistants/clinical officers. A quality antenatal care visit necessitates that medical professionals closely monitor and screen mothers and their babies to identify potential maternal health problems or conditions such as infections, anaemia, and other complications. Appropriate preventive or treatment services can then be provided, thus improving health outcomes for both mothers and newborns. The Ministry of Health’s Clinical Guidelines recommend four antenatal care visits during pregnancy (The Republic of Uganda, 2016). Deliveries in healthcare facilities and, most especially, skilled birth attendance are crucial for reduction of obstetric fistula and infant/maternal mortality. This study used a mathematical model to study the effect of lack of skilled healthcare during child birth in South Central, North Central, Kampala, Busoga, Bukedi, Bugisu, Teso, Karamoja, Lango, Acholi, West Nile, Bunyoro, Tooro, Kigezi and Ankole regions. The model hypothesized that without access to skilled healthcare, pregnant women would likely develop obstetric fistula. The women were grouped into three classes; those with fistula, the ones that accessed treatment for the condition and the recovered. The model was analyzed to determine the best investment strategy; specifically, do we invest in getting women access to skilled healthcare during child birth, or treatment for the affected.

Results show that if we cannot get women access to skilled healthcare during child birth, then there would be no need investing in skilled healthcare. According to the data, births outside of healthcare facilities are much less likely (8%) to be attended by a skilled healthcare provider than births in either public (99%) or private (96%) healthcare facilities. Although almost all women (97%) of child bearing age, 15-49 with a live birth in the past 5 years received skilled healthcare during their most recent pregnancy, only 29% of women had their first healthcare visit during the first trimester of pregnancy and 60% completed the recommended four visits. We need to determine why women do not reach healthcare facilities, and when they do, it is not as prompt as it is medically recommended. Our results also showed that lack of skilled healthcare results in high obstetric fistula rates. Results further showed that provision of skilled healthcare and surgery alone could not stop fistula cases. This leads to conclude that there are other indicators that need to be addressed.

Women in the study gave different reasons why they did not access skilled healthcare such as lack of permission, no money, the long distances to healthcare facilities and lack of support. From the study, the percentage of women who sought skilled healthcare varied by background characteristics. The proportion of births attended by a skilled healthcare provider was lowest in Bunyoro and Bugisu regions (both 58%) and highest in Kampala region (96%). Although there was little variation in receipt of healthcare from a skilled provider by selected background characteristics, the variation in the kind of provider seen was noticeable. Births to urban women are more likely (88%) to take place in a healthcare facility than births to rural women (70%). There was also a large regional variation in the proportion of births in healthcare facilities, from 56% in Bugisu region and 57% in Bunyoro region to 94% in Kampala. It also varied based on whether the women were having their first child or not. Women having the first child were more than twice as likely to see a doctor (13%) as women having their sixth (or later) child (5%). Further, urban women were twice as likely to see a doctor (17%) as rural women (8%). The likelihood of seeing a doctor doubled among women with a secondary education (13%) as compared to women with no education or a primary education (6-7%), and doubled again among women with more than a secondary education (28%). In terms of social status, women in the lower wealth quintiles were less likely (6-8%) to see a doctor than women in the highest wealth quintile (21%). Overall, close to three quarters (74%) of births by the women were delivered by a skilled healthcare provider, and consistent with the pattern observed for skilled healthcare, most births were attended by nurses or midwives (64%). In all, there is a steady rise in skilled assistance during delivery over the past 16 years, from 37% in 2000-01 to 42% in 2006, 58% in 2011, and 74% in 2016.

Looking at the number of women treated after development of fistula, we used two sets of data: data from Kitovu hospital, and data from other fistula centres in Uganda. Data from Kitovu was from 2009 to 2016, while from the other centres we used data from 2000 to 2015. Using regression, we estimated the number of fistula women expected to be treated per one successfully treated woman for both data sets. We also estimated the initial number of women that were treated from each centre. Our results showed that from Kitovu, initially we had 506 women treated, and for every one treated, 8 more would get treatment. The model also estimated that by 2016, Kitovu hospital would be operating 9,396 women. From the other centres, initially 25 women were treated and for every one treated, 7 more would get treatment. In both cases, Kitovu was 3,815 treatments below estimated number while other centres were 1,351 less. This calls for measures to enforce skilled healthcare for all such that we cut costs for treatment of these women. From the data, for example in 2015, minimum number of women treated in Kitovu and other centres was 6,472. Each of these treatments cost $400 [26] for a total amount of $2,588,800. If these funds were diverted to empowering women to account for their health, it could have a larger impact than it did when only 6,472 were operated.

Although provision of skilled healthcare was relatively high, some of the women who reported having had vaginal fistula symptoms had sought some form of treatment. This proportion might however include women seeking care through traditional healers that could do little when modern interventions were needed. Collecting the data had constraints in terms of information about a sensitive and stigmatising disease, and results could underestimate prevalence if a substantial number of women with vaginal fistula did not opt for treatment. This would affect the estimates of point prevalence and corresponding investment strategies. Secondly, only women of reproductive age were interviewed, although women older than age 50 years and those younger than age 15 years could also have fistula. Our estimate for the point prevalence of fistula is slightly lower than previously reported. This difference could have resulted from Uganda’s prioritised maternal health in the national health agenda through the Roadmap for Accelerating the Reduction of Maternal and Neonatal Mortality and Morbidity, and as a strategic and priority healthcare intervention area under the current Health Sector Development Plan. Although uncertain, our estimates could be used to estimate different investment strategies to control obstetric fistula. The model looks at the direct causes and not the indirect causes. These could be considered in future study.

## Supporting information

S1 File Stability analysis. (TEX)
